# Neuroprotective Effect of Neuroserpin in Oxygen-Glucose Deprivation- and Reoxygenation-Treated Rat Astrocytes In Vitro

**DOI:** 10.1371/journal.pone.0123932

**Published:** 2015-04-13

**Authors:** Liang Wang, Yang Zhang, Tetsuya Asakawa, Wei Li, Sha Han, Qinying Li, Baoguo Xiao, Hiroki Namba, Chuanzhen Lu, Qiang Dong

**Affiliations:** 1 Department of Neurology, Huashan Hospital, Fudan University, Shanghai, China; 2 Institute of Neurology, Huashan Hospital, Fudan University, Shanghai, China; 3 Department of Neurology, Drum Tower Hospital, Nanjing University School of Medicine, Nanjing, China; 4 Department of Neurosurgery, Hamamatsu University School of Medicine, Hamamatsu-city, Japan; 5 Department of Psychiatry, Hamamatsu University School of Medicine, Hamamatsu-city, Japan; 6 Department of Neurology, Zhongshan Hospital, Fudan University, Shanghai, China; Univ. Kentucky, UNITED STATES

## Abstract

Neuroserpin (NSP) reportedly exerts neuroprotective effects in cerebral ischemic animal models and patients; however, the mechanism of protection is poorly understood. We thus attempted to confirm neuroprotective effects of NSP on astrocytes in the ischemic state and then explored the relative mechanisms. Astrocytes from neonatal rats were treated with oxygen-glucose deprivation (OGD) followed by reoxygenation (OGD/R). To confirm the neuroprotective effects of NSP, we measured the cell survival rate, relative lactate dehydrogenase (LDH) release; we also performed morphological methods, namely Hoechst 33342 staining and Annexin V assay. To explore the potential mechanisms of NSP, the release of nitric oxide (NO) and TNF-α related to NSP administration were measured by enzyme-linked immunosorbent assay. The proteins related to the NF-κB, ERK1/2, and PI3K/Akt pathways were investigated by Western blotting. To verify the cause-and-effect relationship between neuroprotection and the NF-κB pathway, a NF-κB pathway inhibitor sc3060 was employed to observe the effects of NSP-induced neuroprotection. We found that NSP significantly increased the cell survival rate and reduced LDH release in OGD/R-treated astrocytes. It also reduced NO/TNF-α release. Western blotting showed that the protein levels of p-IKKBα/β and P65 were upregulated by the OGD/R treatment and such effects were significantly inhibited by NSP administration. The NSP-induced inhibition could be significantly reversed by administration of the NF-κB pathway inhibitor sc3060, whereas, expressions of p-ERK1, p-ERK2, and p-AKT were upregulated by the OGD/R treatment; however, their levels were unchanged by NSP administration. Our results thus verified the neuroprotective effects of NSP in ischemic astrocytes. The potential mechanisms include inhibition of the release of NO/TNF-α and repression of the NF-κB signaling pathways. Our data also indicated that NSP has little influence on the MAPK and PI3K/Akt pathways.

## Introduction

Thrombolysis induced by tissue plasminogen activator (tPA) is an important strategy for treating cerebral ischemia (CI). Nevertheless, alongside playing a role as a thrombolytic agent, tPA triggers neurotoxic effects after stroke, including inflammatory damage, excitotoxicity, and basal lamina degradation [1] by enhancing the activation of microglia [2] and neuronal N-methyl-D-aspartate (NMDA) receptor-mediated signaling [3,4]. Neuroserpin (NSP) is an axon-secreted serine protease inhibitor and belongs to the serpins superfamily [5]. It plays a crucial role in cerebral development, neuronal survival, and synaptic plasticity as an inhibition of tPA [5].

Although mechanisms involved in the neuroprotective effects of NSP are poorly understood, it is believed that NSP exerts neuroprotective effects in CI animal models [1,6] and patients [4,7]. The neuroprotective role of NSP in cerebral ischemia is well documented. As early as 2000, Yepes et al. first reported that NSP reduces the volume of ischemic lesions and exhibits neuroprotective effects by protecting neurons from ischemic injury [8]. Cinelli et al. later verified these neuroprotective effects in ischemic mice, showing that NSP reduces activation of microglia [6]. Next, Zhang et al. found adjuvant administration of NSP prolonged the therapeutic window for thrombolysis with tPA [9]. Our previous studies indicated that the reduction of fibrinolytic component and the neuroprotective role of NSP may cause the exacerbation of ischemic injury in diabetic rats [10]. Lebeurrier et al. (2005) reported that NSP prevents NMDA-induced neurotoxicity [11]. A later report from the same laboratory showed that NSP plays crucial roles in the CNS, including the adjustment of neuronal migration, plasticity, and cell death [12]. Wu et al. (2010) found that NSP improved ischemic tolerance and decreased the volume of CI lesions in wild-type and tPA-deficient (tPA^*−/−*^) neurons and mice [13]. Rodriguez-Gonzalez et al. (2011) demonstrated that NSP had neuroprotective effects in CI patients [4] and OGD-treated mixtures of neurons and astrocytes in rats by attenuating tPA-mediated mechanisms of inflammation and disrupting the blood–brain barrier in an ischemic model in vitro [1]. They also found that a high serum NSP level indicates a better outcome in CI patients [7]. Ma et al. (2012) confirmed a dose-dependent neuroprotective effect of NSP in OGD/R rats by inhibiting tPA-mediated acute neuronal excitotoxicity [14]. Moreover, Gelderblom et al. recently reported that an unbalanced expression of NSP and tPA caused a negative outcome in experimental CI, which is believed to be associated with increased microglial activation [5]. These previous clinical and bench studies verified the neuroprotective properties of NSP from different angles, and are helpful in approaching the mechanisms of NSP; however, there have been no studies to date on the effects of NSP on astrocytes in vitro.

Astrocytes are the most numerous cells in the central nervous system (CNS). They play crucial roles in the development and maintenance of CNS by the processing, transfer, and storage of information in the nerves [15]. The role of astrocytes in the ischemic state is complex. Astrocytes interact with neurons by cross-talk, and are crucial in maintaining the survival of neurons in the ischemic brain. Abnormal astrocyte function, induced by ischemia, may have remarkable effects on the survival of neurons [16]. Cerebral ischemia triggers astrocyte activation. It is reported that reactive astrocytes contribute to the protection of the ischemic penumbra, modulate the decrease of the infarct volume, and counteract the effects of edema [17]. Another recent study emphasized the important role of astrocytes in maintaining the function and integrity of blood–brain barrier [18]. Because of the abundance of astrocytes, their wide distribution, and important role in ischemic stroke, we speculate that NSP is neuroprotective to astrocytes. A previous study verified the neuroprotective effect of NSP in ischemic cortical cells (mixture of neuron and astrocytes) [1], the effects of NSP on astrocytes alone remain unverified. The present study had two aims. The first was to confirm whether NSP exerts neuroprotective effects on astrocytes in an in vitro ischemic model. The second was to explore the potential mechanisms of NSP in astrocytes. The influence of inflammatory cytokines along with measurement of proteins related to the NF-κB, MAPK, and PI3K/Akt signaling pathways were also investigated. On the basis of the results of a pre-experiment, we used a NF-κB inhibitor, called sc3060, to verify the cause-and-effect relationship between neuroprotection and the NF-κB pathway.

## Materials and Methods

### Establishment of the OGD astrocytes

#### Preparation of the astrocytes

All the astrocytes were acquired from neonatal Sprague-Dawley rats (age < 24 h, Shanghai Institute of the Chinese Academy of Science, China). All animals were treated as per the National Institute of Health Guidelines for the Care and Use of Laboratory Animals. All experimental procedures were approved by the Animal Care and Use Committee of the Fudan University (authorization No: 072105387). Rats were deeply anesthetized by intraperitoneal injections of sodium pentobarbital (50 mg/kg body weight); their cortical hemispheres were separated from the skull and meninges. Then, the tissues were dissociated by 0.125% trypsin digestion for 10 min at 37°C and gently triturated. The homogenate was placed in 100-mm dishes or 96-well plates at a density of 3–5 × 10^5^ cell/mL and maintained at 37°C in a humidified 5% CO_2_ incubator. Astrocytes were cultured in Eagle’s minimum essential medium (MEM) containing 5.5 mM glucose and 10% fetal bovine serum (FBS) [19], which was replaced every 3 d. At confluence (days 7–9), the astrocyte cultures formed a confluent layer of process-bearing glial fibrillary acidic protein-positive cells (at least 95%).

#### Processes of oxygen and glucose deprivation followed by reoxygenation (OGD/R)

OGD was performed following the processes described in a previous study [20]. Briefly, the original glucose-containing media were removed from all treatment groups and kept aside for future use. Astrocytes were washed three times with deoxygenated glucose-free medium and then incubated in an anaerobic chamber (Model 1025, Forma Scientific, USA) in an atmosphere containing 85% N_2_, 10% H_2_, 5% CO_2_, and <0.1% O_2_ at 37°C. In this study, OGD was continued for 12 h. OGD was terminated by removing the cultures from the chamber and reoxygenation was performed by adding the original media and culturing in a normoxic incubator for 0–24 h at 37°C. The control astrocytes were washed and incubated in a normoxic incubator throughout the common normoxic astrocyte culturing medium (blank control).

### Confirmation of the neuroprotective effect of NSP

#### Administration of NSP

Recombinant human NSP (PeproTech, USA) was diluted with MEM containing 10% FBS 24h before the experiments. NSP was randomly added to the normal astrocyte culturing mediums (100 μL/well) in a 96-well filter plate (AcroPrep, USA). We adjusted NSP solutions to various concentrations (i.e., 0, 1.25, 2.5, 5, 10, 20, 40 ng/mL) and reacted for 1 h, and then subjected to the OGD treatment for 12 h followed by 24 h of reoxygenation (OGD12hR24h). We observed the cell survival rate and relative lactate dehydrogenase (LDH) release of all the OGD12hR24h cells along with a blank control.

#### Confirmation of the efficacy of NSP by measuring the cell survival rate and LDH release

The cell survival rate was calculated as follows: at the end of culture, 3-(4,5-dimethythiazol-2-yl)-2,5-diphenyltetrazolium bromide (MTT, Sigma-Adrich, USA) was added to each well to achieve a final concentration of 0.5 mg/mL. After incubation at 37°C for 4 h, the medium was removed and 100 μL of dimethyl sulfoxide was added to each well and kept for 10 min. The plate was read at 450 nm using a microplate reader (Multiscan MK3; Thermo Labsystems USA). The cell survival rate was calculated as the ratio of the cells survived compared to the blank control.

LDH release was measured following a classic method [21]. Briefly, the medium (50 μL) of each group was transferred from the culture well to a 96-well plate and mixed with 50 μL of reaction using a commercial LDH detecting kit (Sigma, USA). The mixtures were incubated at room temperature in the dark for 30 min, and then 50 μL of stop solution included in the kit was added to each well. After 30 min, the absorbance was read at 492 nm using a microplate reader (Thermo, USA). The maximum LDH release was measured in each well at the end of each experiment, following repeated freezing and thawing. Each experiment was conducted in triplicate, with each experiment containing eight readings. Results are expressed as a percentage of the maximum LDH release, after subtracting background levels determined from the medium alone.

**Fig 1:**
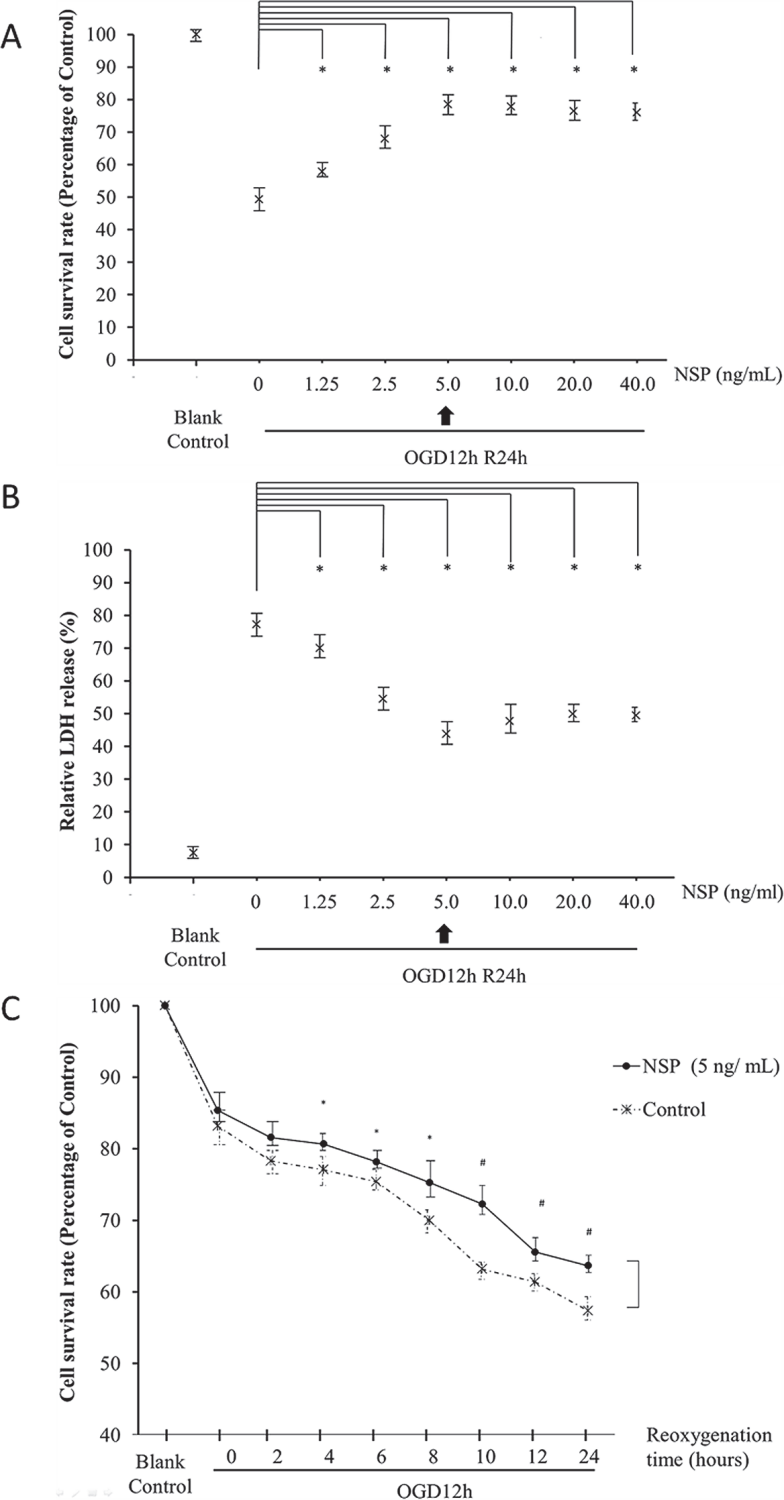
NSP protects astrocytes from OGD-induced injury. Administration of NSP significantly enhances the survival rate of astrocytes (A) and reduces the relative LDH release (B) in OGD12hR24h-treated cells. The administration of 5 ng/mL NSP reached the climax of the protection and improved the cell survival rate of astrocytes treated under different reoxygenation times after 12 h of OGD (C). *P < 0.05, NSP group vs. non-NSP group.

Finally, we selected 5 ng/mL as the appropriate NSP concentration for further experiments (Fig 1A and 1B). We then confirmed the efficacy of 5 ng/mL NSP by measuring the survival rates of cells undergoing 12 h of OGD followed by reoxygenation for various times (i.e., 0, 2, 4, 6, 8, 10, 12, 24 h)

#### Confirmation of the efficacy of NSP by morphological methods

A standard Hoechst 33342 staining was employed to observe changes in DNA in astrocytes, as described in a previous study [22]. Briefly, the astrocytic monolayer solution (8 mL) was fixed using 4% paraformaldehyde (1 h, room temperature). Cells were then stained with 5 μg/mL Hoechst 33342 (Beyotime, China) for 5 min at room temperature in the dark, and washed three times with PBS for 3 min. The morphological features of apoptosis were observed by fluorescence microscopy (Olympus BX 51, Japan), and at least 500 cells from 10 randomly selected fields per dish were counted.

The proportion of apoptotic cells was observed by flow cytometry using the Annexin V-FITC Apoptosis Detection Kit (BioVision, USA), according to a previously described method [23]. Briefly, cells were harvested and resuspended in binding buffer at a concentration of 1 × 10^5^ cells/mL (8 mL). During reoxygenation, cells were simultaneously mixed with annexin V-FITC and propidium iodide (PI). After incubation at room temperature for 10 min in the dark, the cells were analyzed on a flow cytometer (FACScan; BD Biosciences, USA) to determine the population positive for annexin V-FITC (FL-1 channel) and/or PI (FL-2 channel). Data were analyzed using the Cell Quest software (BD Biosciences, USA).

### Exploring the potential mechanisms of NSP

#### The effects of NSP on NO and TNF-α

Enzyme-linked immunosorbent assay (ELISA) was used to observe the influence of NSP on NO and TNF-α. We added the NSP into astrocyte solutions (100 μL) and adjusted the concentration of the NSP solution to 5 ng/mL, and after 1 h of reaction, the cells were treated with OGD12h followed by 12 or 24 h of reoxygenation. The blank control group was not subjected to the OGD/R treatment. Then, NO and TNF-α were measured using the NO ELISA kit and TNF-α ELISA kit (ADL, USA), respectively, according to the manufacturer’s instructions. Nitric oxide was measured as described in a previous study [24]. In brief, we made a standard curve to detect concentrations between 1 and 100 μM of nitrite per well. Next, 100 μL astrocyte solutions were centrifuged at 10,000 g at 4°C for 2 min, and the clarified supernatant recovered. Ice-cold (5%) metaphosphoric acid (Sigma–Aldrich) was mixed with clarified samples in a ratio of 1:1 (v: v) (mixing and spinning at 10,000 g for 5 min). Supernatants along with standards were exposed to nitrate reductase for 1 h at room temperature (23°C) to transform nitrate to nitrite. By using the enhancer, Griess reaction reagents were applied to convert nitrite to a purple azo-cromophore compound. Optical density was measured using a microplate reader (Multiscan MK3; Thermo Labsystems, USA) at 450 nm with a wavelength correction at 570 nm.

#### Verification of the effects of NSP on NF-κB, MAPK, and PI3K/Akt pathways

Classic Western blotting analysis was performed to clarify the potential effect of NSP on the NF-κB [25], MAPK, and PI3K/Akt pathways [26]. We added the NSP into astrocyte solutions (100 μL) and adjusted the concentration of the NSP solution to 5 ng/mL, the cells for the NF-κB pathway were divided into two group. One group was only treated with NSP, and the other group was treated with NSP and the NF-κB inhibitor, sc3060 (Santa Cruz Biotechnology, USA) (75 ug/mL)[27] and after 1 h of reaction, the cells were treated with OGD12 h followed by 24 h of reoxygenation. The blank control group was not subjected to the OGD/R treatment. After OGD/R, the cells were subjected to Western blotting analysis.

Cells were scraped into lysis buffer A (10 mM HEPES, pH 7.4; 10 mM KCl; 0.1 mM EDTA; 0.1 mM EGTA; 1 mM DTT; 1 mM PMSF; 1 μg/mL aprotinin; and 1 μg/mL leupeptin) and centrifuged at 14,000 *g* for 10 min. Supernatants were collected and used as the cytosolic fractions, whereas pellets were resuspended in lysis buffer B (20 mM HEPES, pH 7.4; 400 mM NaCl; 1 mM EDTA; 10% glycerol; 1 mM DTT; 1 mM PMSF; 1 μg/mL aprotinin; and 1 μg/mL leupeptin) on ice for 30 min. These lysates were then centrifuged at 14,000 *g* for 15 min, and supernatants were collected and used the nuclear fractions. Protein concentrations in the resultant supernatants were measured using the BCA protein assay kit (Pierce Manufacturing, USA) and samples containing equal amounts of protein (30 μg) were subjected to electrophoresis on 10% SDS-polyacrylamide gels. After transfer to nitrocellulose membranes, the membranes were incubated in 4% milk in PBS for 1 h at room temperature and then incubated for 12 h at 4°C with antibodies (diluted 1:500) against NF-κB p65, phospho-IKKα/β (p- IKKα/β, CST, USA), phospho-ERK1/2 (p-ERK1/2, CST Co, USA), total-ERK1/2 (t-ERK1/2, Kangchen, China), phospho-AKT (p-AKT, CST Co., USA), total-AKT (t-AKT, Santa Cruz, USA), β-actin (Abcam, USA), and histone H3 (Santa Cruz, USA). The proteins were detected using horseradish peroxidase-conjugated antirabbit or antimouse secondary antibodies (Santa Cruz Biotechnology, USA) and after exposure to a film, they were visualized using chemiluminescence reagents provided with the ECL kit. Densitometry was performed as the index of quantitative analysis using the Quantity One software (BioRad Laboratories, USA).

### Data analysis

**Fig 2:**
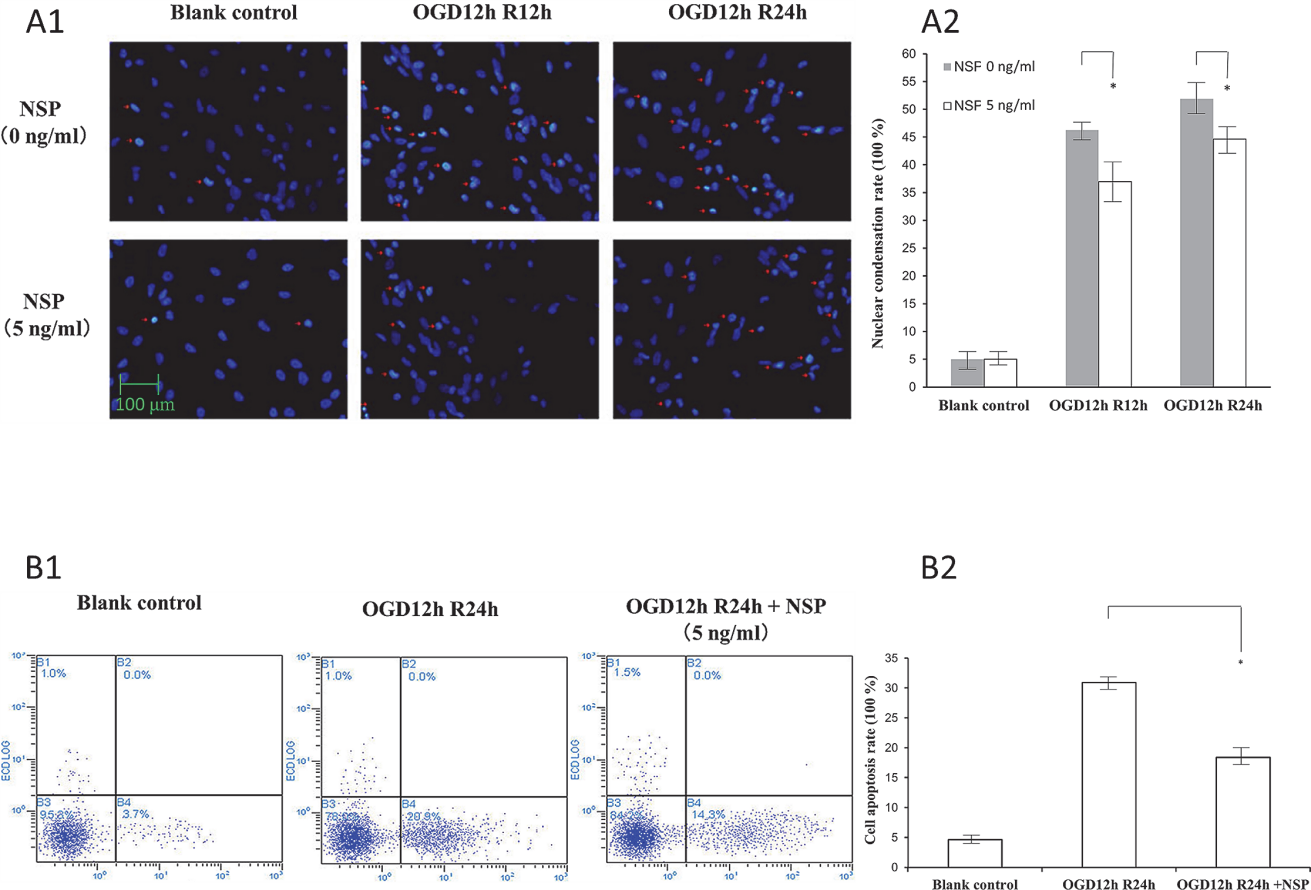
NSP reduces the apoptosis of astrocytes undergoing reoxygenation after OGD (OGD/R). (A) Hoechst 33342 staining. Panel A1 shows the nuclear morphology. The nuclei exposed to OGD/R undergo pyknotic changes (red arrows) and have irregular shapes and these were profoundly reduced in the group treated with 5 ng/mL of NSP (below the line). Panel A2 shows the quantitative analysis of panel A1. The gray columns indicate the control groups (without NSP administration), whereas the white columns indicate the treatment groups administrated with 5 ng/mL of NSP. * P < 0.05, NSP group vs. non-NSP group. (B) Annexin V assay. Panel B1 shows the imaging of OGD12hR24h-treated cells. The number of apoptotic cells (right lower quadrant) increased in the OGD12hR24h group (OGD12hR24h vs. blank control), whereas it reduced in the group treated with 5 nm/mL of NSP (OGD12h24h + NSP vs. OGD12h24h). Panel B2 shows the results of quantitative cell counting: the administration of NSP significantly reduces the cell apoptosis rate. * P < 0.05, NSP group vs. non-NSP group.

All experiments were repeated at least three times in separate cell preparations and the average data were calculated. Data were recorded as the mean ± standard error and were analyzed using the SPSS 13.0.0 software (SPSS, USA). Two-way ANOVA followed by Bonferroni post-hoc correction was used for multiple comparisons. The variables included the NSP dose (Fig 1A and 1B), time of reoxygenation (Fig 1C), reoxygenation time and NSP dose (Figs 2A2 and 3), NSP dose and OGD (Figs 4B and 4C and 5B and 5C). The type I error in ANOVA was accepted as P < 0.05. All the tests were two-sided.

**Fig 3:**
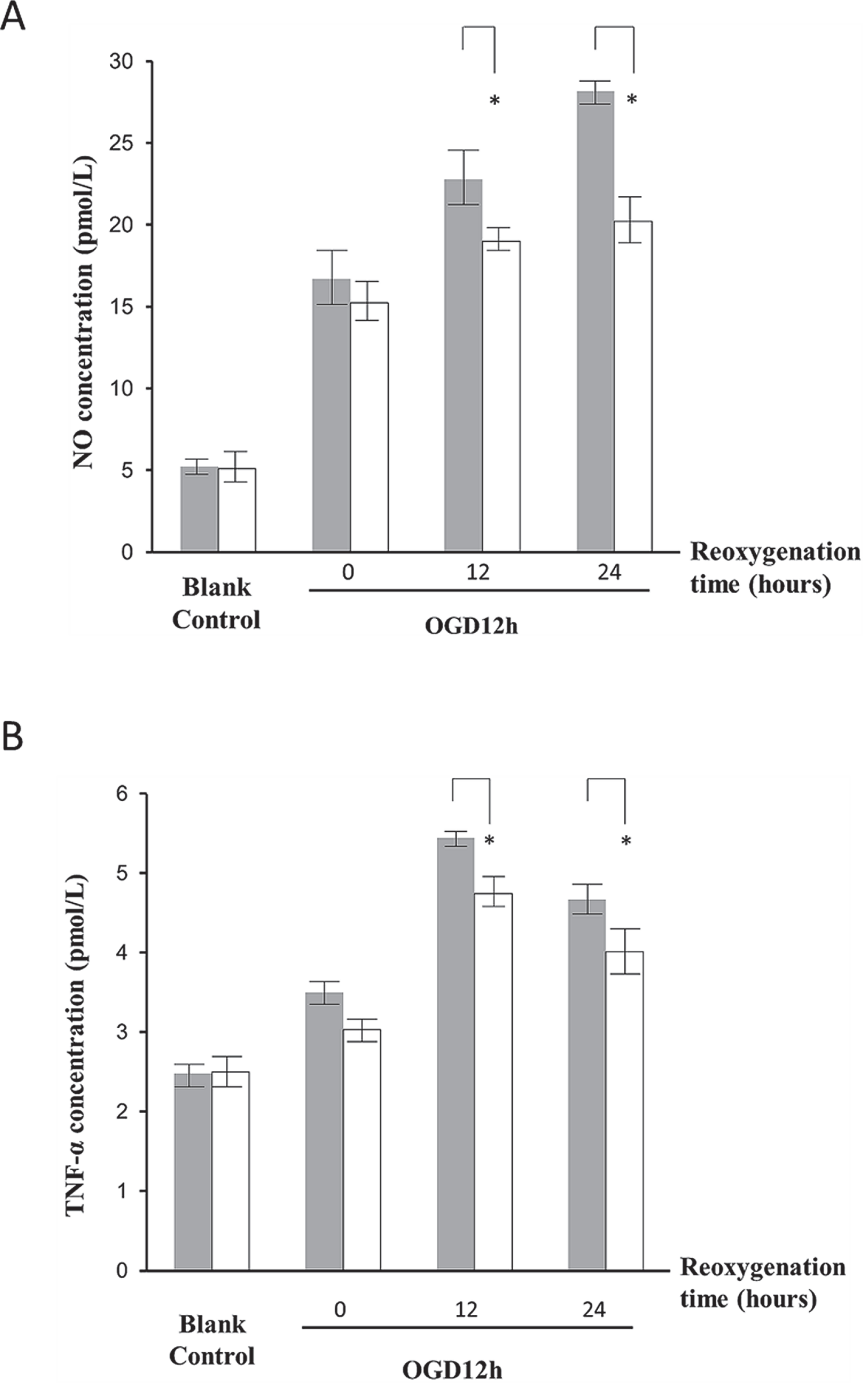
NSP inhibits the release of NO and TNF-α. The releases of NO (A) and TNF-α (B) from the OGD12h-treated cells after 12- and 24-h of reoxygenation were significantly reduced by NSP administration. The gray columns indicate the control groups (without NSP administration), whereas the white columns indicate the groups treated with 5 ng/mL of NSP. * P < 0.05, NSP group vs. non-NSP group.

**Fig 4:**
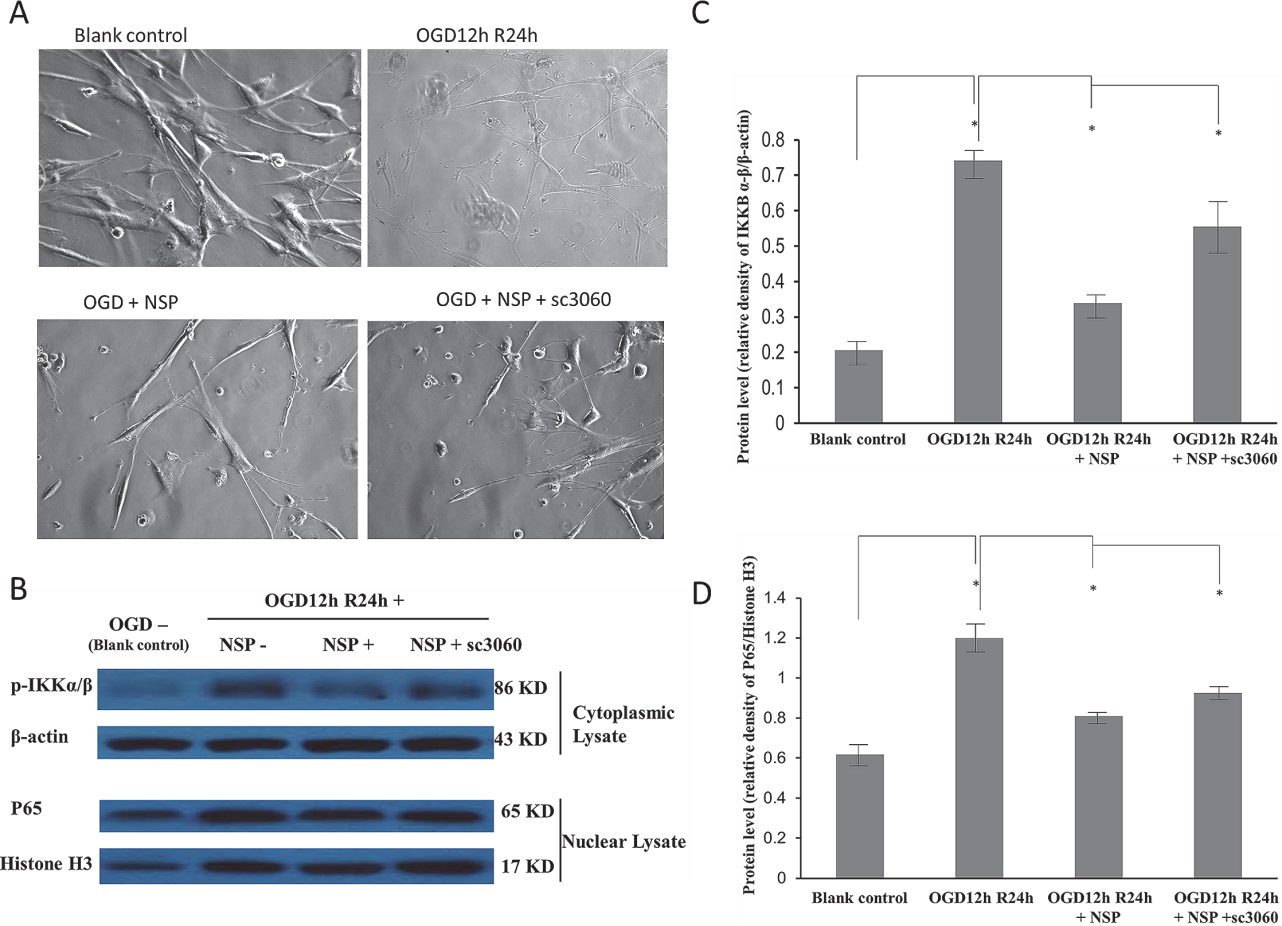
Effects of NSP and the NF-κB inhibitor, sc3060. A. The morphological changes induced by OGD, NSP, and NSP + sc3060 processes. Typical star-shaped astrocytes could be observed in the blank control group. No typical astrocytes were observed in the OGD group. We did observe typical star-shaped astrocytes in the OGD12hR24h + NSP group, but not if we added the NF-κB inhibitor, sc3060. B, C, D showed Western blotting analyses of the cytoplasmic lysate and nuclear lysate in the OGD 12h and R24h-treated astrocytes. β-Actin was used as the loading control of the cytoplasmic lysate and histone H3 of the nuclear lysate. (B) p-IKKBα/β (86 kD) was upregulated in OGD 12h and R24h-treated cells (the second array), but downregulated by NSP administration (the third array) and such downregulation was arrested by the NF-κB inhibitor, sc3060 (the fourth array). Analogously, the expression of P65 (the third line, 65 kD) was upregulated in OGD12h and R24 h-treated cells (the second array), but was downregulated by NSP administration (the third array), and such downregulation was again arrested by NF-κB inhibitor, sc3060 (the fourth array). The protein levels of p-IKKBα/β (B) and P65 (C) were enhanced by the OGD 12h and R24h treatment (OGD 12hR24 h vs. blank control), whereas NSP administration significantly inhibited such effects (OGD 12hR24 h vs. OGD12 hR24 h + NSP). The inhibition by NSP significantly recovered after administration of NF-κB inhibitor, sc3060 (OGD12hR24h + NSP vs. OGD12hR24h + NSP + sc3060),* P < 0.05.

**Fig 5:**
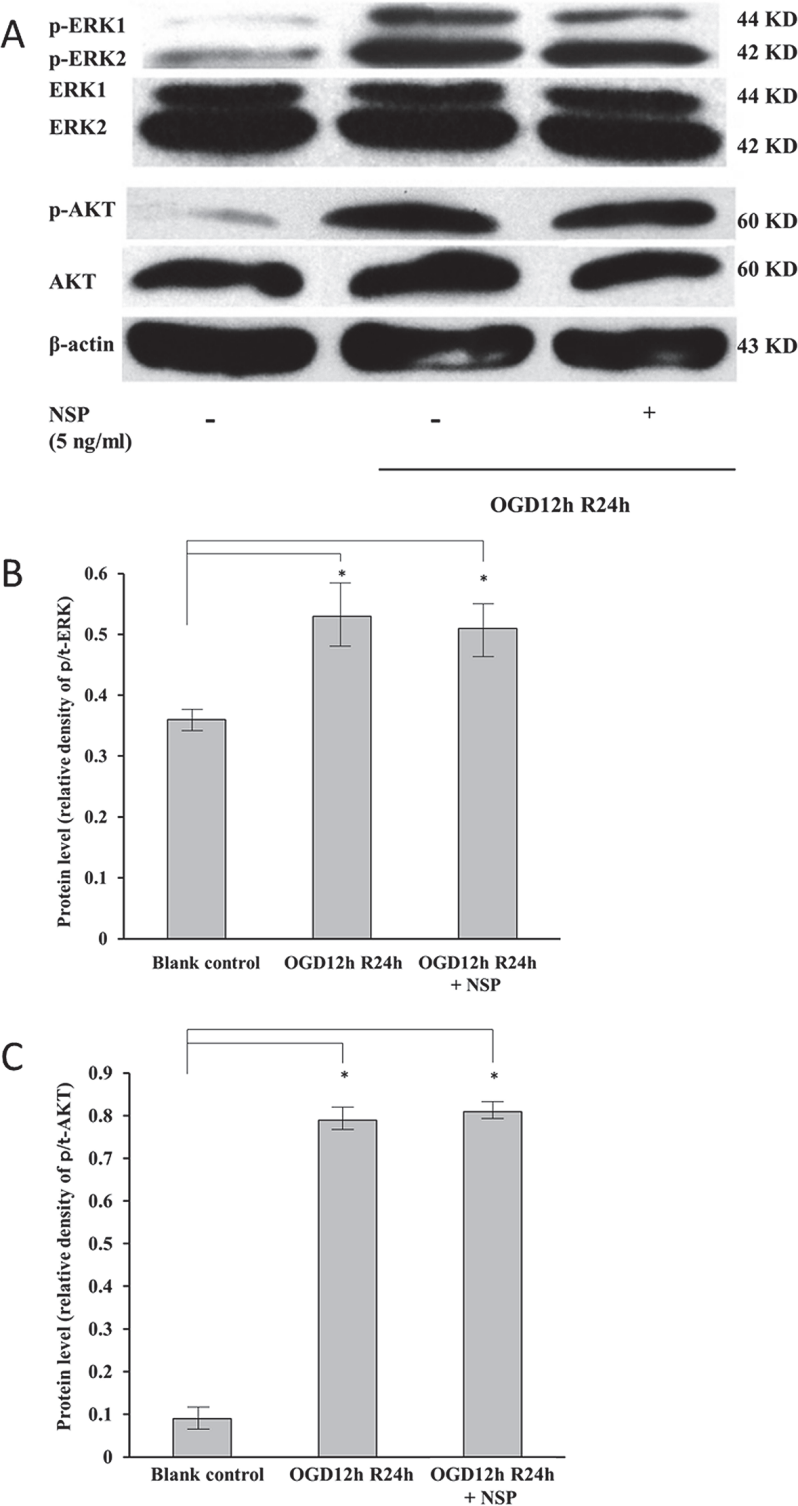
Western blotting analyses of ERK1, ERK2, and Akt in the cells treated with OGD12hR24h and NSP. Total expressions of t-ERK1 (the third line, 44 kD), t-ERK2 (the fourth line, 42 kD), and t-Akt (the sixth line, 60 kD) were used as the loading controls. (A) The expressions of p-ERK1 (the first line, 44 KD), p-ERK2 (the second line, 42 KD), and p-AKT (the fifth line, 60KD) were upregulated by treatment with OGD12hR24h (the second array), and were unchanged by NSP administration (the third array). The protein levels of p/t-ERK (B) and p/t-AKT (C) were significantly enhanced by the OGD12hR24h treatment (OGD12hR24h vs. blank control, n = 12, P < 0.05), and NSP administration does not have any affect (OGD12hR24h vs. OGD12hR24h + NSP). * P < 0.05.

## Results

### Confirmation of the neuroprotective effects of NSP

#### Protection of the OGD-induced injury by NSP administration

Fig 1 shows the protective effects of NSP on the astrocytes treated by OGD/R. The survival rate of the cells was significantly reduced after OGD12hR24h (NSP 0 ng/mL vs. blank control); however, the administration of NSP significantly increased the survival rate. The maximum effect was observed with 5 ng/mL of NSP, but no further improvements were observed at a dose over 5 ng/mL (Fig 1A).

The relative LDH release of the cells significantly increased after OGD12hR24h (NSP 0 ng/mL vs. blank control); however, the administration of NSP significantly decreased the LDH release. The maximum effect was observed with 5 ng/mL of NSP, but no further improvements were observed at a dose over 5 ng/mL NSP (Fig 1B).

Thus, we selected 5 ng/mL as the administration dose for further experiments.

We also found that 5 ng/mL of NSP improved the cell survival rate in the cells treated by OGD12h followed by different times of reoxygenation (4–24 h, Fig 1C).

#### Inhibition of the OGD/R-induced apoptosis by NSP

Fig 2 shows the protective effects of NSP by morphological methods.

Fig 2A shows the results of DNA staining (Hoechst 33342 staining). By comparing the number of pyknotic changes between the non-NSP group and NSP group, it is obvious that NSP administration inhibited the changes induced by OGD/R (Fig 2A1). The results of quantitative counting are shown in Fig 2A2.

Fig 2B shows the results of the Annexin V assay. The apoptotic cells increased by OGD/R treatments (OGD12h24h vs. blank control, right lower quadrant), whereas NSP administration exerted an inhibitory effect against ischemia-induced apoptosis (OGD12h24h vs. OGD12h24h + NSP, Fig 2B1). Fig 2B2 shows the results of quantitative cell counting.

### The exploration of the relative mechanisms

The potential mechanisms of neuroprotection were investigated by measuring the release of NO and TNF-α and the proteins related to the NF-κB, MAPK, and PI3K/Akt pathways.

#### Inhibition of the release of NO and TNF-α by NSP

Fig 3 shows that NSP inhibited the release of NO (Fig 3A) and TNF-α (Fig 3B). Inhibition of NO and TNF-α may play a role in the mechanism of NSP neuroprotection.

#### Inhibition of the NF-κB pathway by NSP

Fig 4 shows the results of the morphological studies and Western blotting analysis used to assess the activation of the NF-κB pathway, including expression of p-IKKα/β and p65.

We observed typical star-shaped astrocytes with synapses enwrapped in a slice of blank control, but the typical astrocytes disappeared after OGD 12 h and R24 h. Administration of NSP was neuroprotective, as we observed characteristic star-shaped astrocytes in the slice, but these neuroprotective effects could be reversed by administration of the NF-κB pathway inhibitor, sc3060.

For the cytosolic fractions, the expression of p-IKKα/β was significantly enhanced by the OGD12hR24h treatment and was recovered after the administration of NSP. The recovery induced by NSP could be reversed by administration of the NF-κB pathway inhibitor, sc3060 (Fig 4B and 4C). For the nuclear fractions, the expression of nuclear NF-κB p65 was upregulated by the OGD12hR24h treatment, whereas it reduced significantly after the administration of NSP. The reduction induced by NSP could be reversed by administration of sc3060 (Fig 4B and 4D). These results indicated that inhibition of the NF-κB pathway may play a role in the mechanism of NSP neuroprotection.

#### NSP has little influence on the MAPK and PI3K/Akt pathways

Fig 5 shows that, although the OGD12hR24h treatment upregulated the expressions of p-ERK1, p-ERK2 (Fig 5A and 5B), and p-Akt (Fig 5A and 5C), interestingly, the administration of NSP did not invert that trend, which indicated that NSP has little influence on the MAPK and PI3K/Akt pathways.

## Discussion

Thrombolysis with tPA is the most common therapy for treating CI, despite tPA administration being a double-edged sword. In this regard, a deeper insight into the tPA inhibitor NSP, which plays a crucial role in tPA regulation [12,28], is particularly important. Although the neuroprotection of NSP on cerebral ischemia is well documented, the present study aimed to confirm the neuroprotective effects of NSP on ischemic astrocytes in vitro and attempted to explore the related mechanisms. To the best of our knowledge, this is the first study focusing on the mechanisms of the effects of NSP on astrocytes.

Early in 1999, Docaqne et al. reported that NSP is expressed in both neurons and astrocytes (star-shaped glial cells in CNS) [29]. Our findings verified the neuroprotective effects of NSP on astrocytes i.e., NSP administration significantly reduced the release of LDH (Fig 1B) in OGD/R-treated astrocytes. In addition, the cell survival rate (Fig 1A) along with the morphological evidences (Figs 2 and 4A) confirmed the neuroprotective effects of NSP on astrocytes. These findings are in agreement with the results of Rodriguez-Gonzalez et al. [1]. In that study, they found that OGD treatment caused morphological alterations and increased cell death in these cells, whereas the administration of NSP could reverse these changes [1]. The results of our present study are different on two accounts. First, their study, which involved a mixture of neurons and astrocytes, did not focus on the mechanisms underlying the effect of NSP on astrocytes. Second, they used the OGD process, whereas in this study, we used the OGD/R process in order to mimic the ischemic state of astrocytes [1,14,20]. All of our evidence proved that NSP is neuroprotective to astrocytes under ischemic conditions.

**Fig 6:**
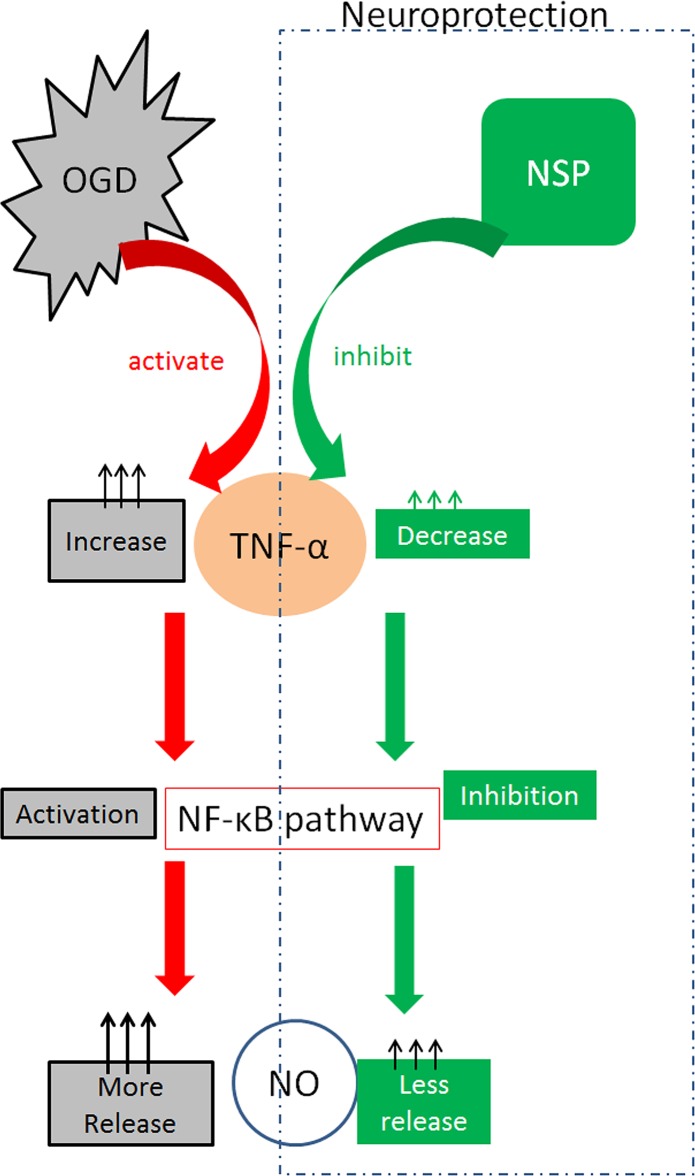
Potential mechanisms for NSP neuroprotection via the NF-κB pathway. Red arrows indicate activation. Green arrows indicate inhibition.

The mechanism of NSP neuroprotection is complicated. Previous studies concerning NSP focused on its effects in neurodegenerative disorders such as Alzheimer’s disease [25,30–32], and most of the mechanisms were discussed with regard to its neuroprotective effects in NMDA-induced cell death [3,4,29] and microglia [2,5,33]. Previous studies have suggested that NSP may regulate the growth and maturation of neurons via a noninhibitory mechanism [28,34]. In the present study, once the protective efficacy was verified, we explored other potential neuroprotective mechanisms. The first finding was that NSP could inhibit the release of NO and TNF-α (Fig 3). Oxidative and inflammatory pathways have been proven to play roles in the injuries induced by OGD/R exposure [20]. The release of NO and TNF-α can be used as a simple biomarker in the future. The second finding was that NSP administration considerably reduced the overactive expression of p-IKKα/β and P65 in OGD/R astrocytes (Fig 4B and 4D). The protection induced by NSP could be significantly reversed by sc3060, a NF-κB pathway inhibitor. We first reported that blocking the NF-κB pathway deteriorates the morphological appearance of astrocytes ameliorated by NSP (Fig 4A), and reverses the downregulation of p-IKKα/β and P65 expression induced by NSP (Fig 4B and 4D). These data suggest the critical role of the NF-κB pathway in NSP-induced neuroprotection in cerebral ischemia. The NF-κB pathway acts as an important transcription factor and regulator of genes that control cell proliferation and survival. It is regarded as the center of various cellular responses, including immune and inflammatory reactions [35], and is generally considered as a “prototypical proinflammatory” signaling pathway on the basis of the activation of NF-κB by proinflammatory cytokines [36]. The roles of the NF-κB pathway under ischemic conditions are dual, and the interaction between the NF-κB pathway and inflammatory cytokines such as TNF-α is complicated. Nijboer et al. reported that early NF-κB activation is neurotoxic, whereas the late NF-κB pathway is neuroprotective. Inhibition of the early NF-κB pathway is neuroprotective only if the late NF-κB pathway is maintained [37]. It is believed that TNF-α can directly activate the NF-κB pathway, and such activation of the NF-κB pathway may generate NO by stimulation of the expression of nitric oxide synthase [38]. The interaction between NSP and the NF-κB pathway is poorly understood. It is believed that NF-κB may be activated by the accumulation of NSP polymers within the lumen of the endoplasmic reticulum. Chronic activation of NF-κB may cause cell death, resulting in the accumulation of NSP and causing dementia [39]. On the basis of the above evidence, we speculate that a potential explanation of our findings is that in the early stage of ischemic/hypoxic injury to astrocytes (OGD12h), administration of NSP inhibits the release of TNF-α, which causes less activation of the NF-κB pathway and consequent reduction in the release of NO (Fig 6). This should be verified in our next experiments. One limitation of this study is that we did not measure the changes in NO after administrating sc3060. However, astrocyte morphology (Fig 4A) provides evidence that NF-κB pathway inhibitor can reverse the efficacy of NSP. Another limitation is that we did not have a control condition, i.e. only with OGD, in all our experiments. We aim to do this in future experiments.

Moreover, we also found that the ERK1/2 and PI3K/Akt signaling pathways were activated by the OGR/R treatment (Fig 5), which was in accordance with a previous study [40]. However, our data showed that NSP has only little influence on these pathways. Additional studies are warranted to clarify the interactions between these complicated pathways and NSP.

## Conclusions

In conclusion, NSP exerts neuroprotective effects in OGD/R-treated astrocytes, and these potential neuroprotective mechanisms may lie in the inhibition of release of TNF-α and NO, along with inhibition of the NF-κB pathway. In addition, NSP has little influence on the MAPK and P13K/Akt pathways.
